# Characterisation of a novel cell line (CSQT-2) with high metastatic activity derived from portal vein tumour thrombus of hepatocellular carcinoma

**DOI:** 10.1038/sj.bjc.6605689

**Published:** 2010-05-11

**Authors:** T Wang, H S Hu, Y X Feng, J Shi, N Li, W X Guo, J Xue, D Xie, S R Liu, M C Wu, S Q Cheng

**Affiliations:** 1 Eastern Hepatobiliary Surgery Hospital, Second Military Medical University, Shanghai, China; 2 Laboratory of Molecular Oncology, Institute for Nutritional Sciences, Shanghai Institutes for Biological Sciences, Chinese Academy of Sciences, Shanghai, China; 3 Changhai Hospital, Second Military Medical University, Shanghai, China

**Keywords:** PVTT, HCC, IVIS, TIC, cell line, metastasis

## Abstract

**Background:**

Portal vein tumour thrombus (PVTT) is highly associated with the progression and metastasis of hepatocellular carcinoma (HCC). However, there are no appropriate cell models of PVTT with which to study the biological and physiological characteristics of PVTT.

**Methods:**

Primary cell culture was performed by the use of a successive xenograft line called PVTT-#1, which was obtained from a 60-year-old male HCC patient accompanied by PVTT.

**Results:**

A successive cell line named CSQT-2 was established. The cell line showed aggressive phenotypes in terms of cell growth, survival, migration, xenograft and metastasis. Moreover, an orthotopic transplantation assay showed that PVTT can be generated in nude mice when CSQT-2 cells were inoculated in the liver and that it shows a typical migratory tendency in the vascular branches of portal vein. Moreover, the established CSQT-2 cells also showed varied expression of tumour-initiating cell (TIC) markers such as CD133, CD90 and EpCAM.

**Conclusion:**

Establishment of CSQT-2 may provide a suitable model with which to investigate the molecular mechanisms of PVTT-related HCC.

Hepatocellular carcinoma (HCC) ranks as the fifth most common cancer around the world, (Parkin, 2006; Portolani *et al*, 2006; Yamane and Weber, 2009), and it is one of the major health problems worldwide. Currently, the geographical areas bearing the highest incidence are eastern Asia as well as middle and western Africa, whereas the incidence is increasing in North and South Americas (Bosch *et al*, 2005; Michielsen *et al*, 2005; Chen *et al*, 2006). In addition, the mortality due to liver cancer has shown the fastest rate of increase in the United States, even though overall cancer-related mortality has been declining (www.seer.cancer.gov). In fact, liver cancer metastasis and recurrence are known to be highly lethal (Kornek *et al*, 2008). Unfortunately, the poor understanding of the development of HCC metastasis has been the main obstacle for the improvement of HCC therapy (Shuqun *et al*, 2004; Sun *et al*, 2007; Amarapurkar *et al*, 2008; Lim *et al*, 2008; Ramaiah and Rittling, 2008). Portal vein tumour thrombus (PVTT) arising from the invasion of HCC cells into the portal vein, is a special type of HCC metastasis (Shuqun *et al*, 2007; Zhou *et al*, 2008). Chau *et al* (2003) has reported that the incidence of microscopic tumour thrombi is about 49.6%. As a well-known poor prognostic factor, PVTT markedly deteriorates the hepatic function and correlates with greater recurrence and intrahepatic metastasis (Li *et al*, 2009). Nevertheless, the aetiology of PVTT in HCC metastasis is largely unknown (Shuqun *et al*, 2006; Inoue *et al*, 2009; Inui *et al*, 2009). The lack of an experimental model may be responsible for our poor understanding of the mechanism through, which PVTT is involved in HCC metastasis (Takizawa *et al*, 2007; Liang *et al*, 2008). However, in many cases, the primary cultures of PVTT cells are not able to survive and thrive for a long time (Choi *et al*, 2008). Therefore, the aim of this study was to establish a PVTT-originated HCC cell line that has characteristics of PVTT. We have successfully developed a human PVTT-originated xenograft model in nude mice, named PVTT-#1 (submitted for publication), which has thrived for generations. Here, by using the orthotropic tumour of this model, we succeeded in establishing novel PVTT-originated HCC cell lines that could contribute to the study of PVTT.

## Materials and methods

### Experiment design

This experimental protocol was approved by the Institutional Review Board of the Eastern Hepatobiliary Hospital. Informed consent was obtained from the patient.

### Patient

Tumour tissue was obtained from a 60-year-old male patient from northwest China who was diagnosed with HCC accompanied by PVTT. His oncological family history was unknown. He had no history of operations. Blood tests showed an AST level of 86 U l^–1^, *α*-fetoprotein level of 258 ng ml^–1^, carcinoembryonic antigen level of 2.8 ng ml^–1^ and positivity for HBsAg. Before operation, the patient complained of tender upper right abdominal pain. A computerised-tomography showed a lesion (10 cm × 11 cm) in the right lobe of the liver and a PVTT invasion to the right branch of the portal vein. We have performed a partial liver resection plus thrombectomy on the patient. Pathological evaluations confirmed that both HCC and PVTT showed mid-level differentiation on the Edmondson criteria and that the liver cirrhosis was mild. The patient died of lung metastasis 3 months after hepatectomy.

### Animals and cell lines

We obtained 6-week-old male BALB/c nude mice from the Institute of Nutritional Sciences, Chinese Academy of Science, and they were maintained in a specific pathogen-free condition. The study protocol on mice was approved by the Shanghai Medical Experimental Animal Care Commission. The 7702 liver cancer cell lines were purchased from the Cell Bank of Type Culture Collection of the Shanghai Institute of Cell Biology, Chinese Academy of Sciences. They were cultured in RPMI 1640, and supplemented with 10% fetal bovine serum, 10 units ml^–1^ penicillin, and 10 units ml^–1^ streptomycin, at 37°C in a humidified atmosphere containing 5% CO_2_. MHCC97-H and MHCC97-L cell lines were purchased from the Liver Cancer Institute of Fudan University, Shanghai. They were cultured in DMEM and supplemented with 10% fetal bovine serum, 10 units ml^–1^ penicillin and 10 units ml^–1^ streptomycin.

### Antibodies and reagents

Rabbit anti-human phospho-Akt and EGFR antibodies were brought from Cell Signal. Mouse monoclonal anti-human *β*-Actin antibody was purchased from Santa Cruz Biotechnology. APC-labelled anti-CD133 and FITC-labelled anti-CD90, EpCAM antibodies were brought from MACS.

### Establishment of the PVTT xenograft model

The establishment of the PVTT xenograft model was performed as described by Sun *et al* (1996), with slight modifications. We initially developed a successive tumour line in nude mice using the patient's portal vein tumour thrombus specimen. When the patient's PVTT specimen was obtained, it was cut into pieces with dimensions of about 2 mm × 2 mm × 2 mm that were implanted into the liver of each of the six nude mice. Once the orthotopic tumour was able to survive and developed into a gross tumour bulk, it was removed and cut into pieces of about 2 mm × 2 mm × 2 mm in dimension, which were implanted into the liver and subcutaneously (in the armpit and groin) of each nude mouse, using the method described previously. At last, we succeeded in establishing the xenograft model named PVTT-#1.

### Culture of primary and successive cell lines

Several attempts at primary cultures were made using the orthotropic tumour derived from the PVTT-#1 xenograft model described above. These xenografts were removed and used for primary culture *in vitro* by different culture methods as described by Tian *et al* (1999). In the end, we observed that the most suitable environment for cell culture was in DMEM with 10% fetal bovine serum at 37°C in a humidified atmosphere with 5% carbon dioxide *in vitro*. The successive HCC cell line that passed more than 100 generations was named as CSQT-2.

### RNA Extraction and RT PCR assay

Total RNA was isolated with TRIzol reagent (Invitrogen) from CSQT-2 cell lines and from tissues of PVTT patients after their informed consent was obtained according to institutional regulatory requirements. The RNA samples were separated in 2% agarose gels containing ethidium bromide, and their quality was then determined by visibility of the 18 S and 28 S RNA bands under UV light. A total of 2 *μ*g of high quality RNA was processed directly to cDNA with the reverse transcription kit (Promega, Madison, WI, USA), following the manufacturer's instructions. The PCR procedure used for the detection of human cIAP1, EGFR, Integrin *β*-5, uPAR, cMet and *β*-actin was slightly modified from the procedure described by Tian *et al* (1999).

### DNA isolation

Cultured cells (1 × 10^6^) were thoroughly homogenised in 400 *μ*l DNA denature solution, then incubated in 100 *μ*l 5% SDS (m V^–1^) buffer containing 20 *μ*l RNAase and 20 *μ*l proteinase K at 37°C for 24 h. The DNA was extracted with phenol–chloroform. Extracted DNA was precipitated with ethanol and resuspended in distilled water.

### Cytogenic analysis

Chromosome preparation and G-banding were performed as Seabright (1971) described with slight modifications. Briefly, colcemid was added to cell cultures at the 60th passage to a final concentration of 0.25 mg ml^–1^ for 10–15 h. Cells were then trypsinised and centrifuged at 1200 r.p.m. for 12 min. The hypotonic medium used was 0.075 mol potassium chloride for 20 min at 37°C, and then the fixative (methanol/glacial acetic acid, 3 : 1) was changed three times before slides were made. Some of the slides were aged at 60°C for 8–10 h for G-banding. The aged slides were trypsinised for 3.5 min, rinsed in water and stained with Giemsa, rinsed again, air dried and examined under the microscope. For C-banding, the rest was soaked in 0.2 N hydrochloric acid for 1 h, and then incubated in 5% Ba(OH)2 at 56°C for 10 min and rinsed in water. After that, the slides were re-incubated with 2 × SSC (saline sodium citrate) at 67°C for 1–1.5 h, rinsed again and stained with Giemsa.

### Western Blot

Cultured cells were washed twice with PBS and lysed in RIPA buffer for 30 min on ice. Cell lysates were clarified by centrifugation at 10 000 g for 15 min, and the protein concentration was determined using the Bradford reagent (Sigma). Total cell lysates (40 mg protein) were separated using 10% SDS–PAGE gels and electrotransferred onto polyvinylidene difluoride membranes (Millipore, Bedford, MA, USA). After blockage with 5% milk, the membranes were probed with primary antibodies at 1 : 1000 dilutions. The membrane was washed and then incubated with horseradish peroxidase-conjugated secondary antibodies for 1 h. Immunoreactive proteins were detected with enhanced chemiluminescence reagents (Pierce, Rockford, IL, USA) and photographed with Kodak X-Omat blue autoradiography film.

### Cell surface marker expression analysis

Cells (10^6^) were trypsinised and suspended in 100 *μ*l D-Hank's buffer. APC or FITC-labelled cell surface marker-targeted antibodies (CD133, CD90 and EpCAM) (10 *μ*l) were added and cells were incubated on ice for 20 min. Then cells were washed with D-Hank's buffer twice, re-suspended in 300 *μ*l D-Hank's buffer and sent for FACS analysis.

### MTT assay

The assay of the cell growth curve was performed as Patterson Jr MK (1979) described, with slight modifications. Cells were plated into 96-well plates at 3000 cells per well, cultured in 10% FBS DMEM for various durations, and the cell numbers were measured by MTT assay according to the protocol provided by the MTT manufacturer.

### Soft agar assay

Cells were plated in 24-well flat-bottomed plates using a two-layer soft agar system with 1 × 10^3^ cells per well in a volume of 400 *μ*l per well as described previously (Munker *et al*, 1986). After 14 days of incubation, colonies were counted and measured. All the experiments were repeated at least three times using triplicate plates per experimental point.

### Boyden chamber

Boyden chamber (8-*μ*m pore size polycarbonate membrane) was obtained from Neuroprobe Corp, Bethesda, MD, USA. Cells (2 × 10^5^) in 0.05 ml medium with 1% FBS were placed in the upper chamber, and the lower chamber was loaded with 0.152 ml medium containing 10% FBS. Cells that migrated to the lower surface of filters were detected with traditional H&E staining, and five fields of each well were counted after 4–24 h of incubation at 37°C with 5% CO_2_. Three wells were examined for each condition and cell type, and the experiments were repeated thrice.

### Tumourigenicity assay

Cultured cells of CSQT-2 were washed with D-Hank's solution, and 100 000, 10 000 or 1000 cells suspended in 100 ml of DMEM and 100 ml Matrigel were injected into the flanks of 6-week-old male nude mice purchased from the Shanghai Laboratory Animal Centre, CAS (Shanghai, China). They were treated in accordance with the American Association for the Accreditation of Laboratory Animal Care guidelines. Three mice were used in the groups with different cell numbers. The resulting tumours were measured once a week before the mice were killed and the tumour volume (mm^3^) was calculated using the standard formula: length × width × height × 0.5236.

### Orthotopic transplantation

For orthotopic transplantation, obtained specimens were cut into pieces of about 2 mm × 2 mm × 2 mm in dimension that were implanted into the liver and subcutaneously (in the armpit and groin) of each of the six nude mice, using the method as described by Sun *et al* (1996) mentioned above. Once the subcutaneous tumour reached 1–1.5 cm in diameter, it was removed and cut into pieces of about 2 mm × 2 mm × 2 mm in dimension that were implanted into the liver and subcutaneously (in the armpit and groin) in each of six nude mice, using the methods described previously.

### Luciferase labeling of CSQT-2 cells

Luc2 luciferase-coding region sequence was amplified by PCR and cloned into the FG12 lentiviral expression vector. FG12-Luc2 construct was transfected into packaging-293T cells for lentivirus production. CSQT-2 cells were infected by the FG12-Luc2 lentivirus to stably express luciferase gene.

### Experimental metastasis assay

For the experimental metastasis assay, two routes of injection were used in our experiment. The intracardiac and the intraspleen routes. A total of 500 000 cells were used in each injection. The metastatic tumour growth was measured by luciferase activity detection using the IVIS. Male nude mice were housed under standard conditions. For intracardiac injections, subconfluent cells were harvested, washed in PBS and resuspended at a concentration of 1 × 10^7^ cells per ml. BALB/c nude mice were anaesthetised by intraperitoneal injection of Chloral hydrate (30 mg kg^–1^) and were placed in the supine position. After visualisation of arterial blood flow into the syringe, 5 × 10^5^ cells were injected into the left ventricle via the third intercostal space. Successful injections were confirmed by immediate IVIS. For intrasplenic injections, anaesthetised mice were placed in the supine position on the nude mice tables. The spleen of the nude mouse was explored with skin and peritoneal incisions, and 5 × 10^5^ cells of CSQT-2 were injected into the spleen of nude mice for metastasis. Metastasis appeared 4 weeks after injection. Successful injections were confirmed by immediate IVIS. All animal studies were performed in accordance with the SIBS Guide for the Care and Use of Laboratory Animals and approved by the Animal Care and Use Committee, Shanghai Institutes for Biological Sciences.

## Results

### Establishment of a HCC cell line

To establish a HCC culture, we first developed a successive tumour line in nude mice using the patient's portal vein tumour thrombus specimen mentioned above. Next, a primary culture was made using the orthotropic tumour derived from that tumour line. At last, we succeeded in maintaining the primary cultured cells and subculturing them for more than 100 generations in DMEM with 10% fetal bovine serum *in vitro*. This successive HCC cell line was named as CSQT-2.

### Morphology of the HCC cell line CSQT-2

**Figure 1:**
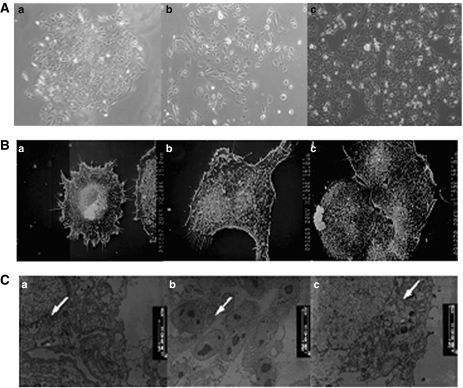
Morphology of CSQT-2 cell line. (**A**) Microphotograph of the cultural cell line CSQT-2 of different passages in culture. a: primary cell, b: Passage 3, c: Passage 55. (**B**) Scanning electron microscopy of CSQT-2 cells showing microvilli on cell surface (s.e.m., × 2000). (**C**) Transmission electron microscopy of CSQT-2 cell line displaying irregular desmosomes, mitochondrium, ribosomes and nuclei. (TEM, × 7 500).

In the initial few passages, CSQT-2 cells formed intact colonies showing typical epithelial cell shapes (Figure 1A). With successive passages the CSQT-2 cells still retained their epithelial cell shapes, but they moved away from each other and lost the colony type formation compared with the initial passage (Figure 1A). Ultrastructurally, the CSQT-2 cells showed intense microvilli on the cell surfaces, which were probably formed by the dysregulation of actin and tubulin-based cytoskeleton (Figure 1B). Upon examination with the transmission electron microscope, most of the CSQT-2 cells were found to contain irregular desmosomes, mitochondrium, ribosomes and nuclei (Figure 1C).

### Characteristics of CSQT-2 cell line *in vitro*

**Figure 2:**
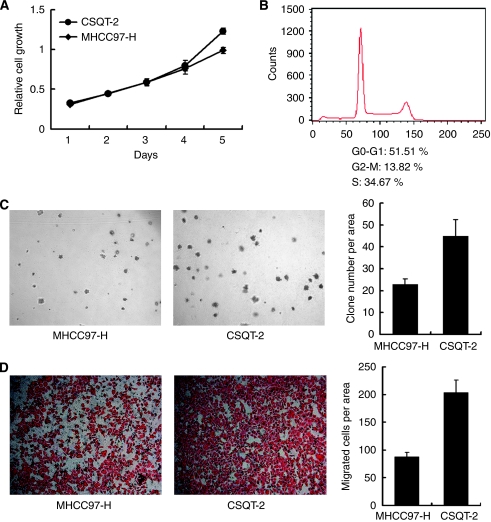
*In vitro* assay of CSQT-2 cell line. (**A**) Cell growth of CSQT-2 and MHCC97-H. Both cells were plated in 96-well plate with a concentration of 3000 cells per well. MTT assay was performed each day to depict a growth curve. (**B**) Cell cycle analysis of CSQT-2. CSQT-2 cells were harvested for FACS analysis after PI staining. (**C**) Anchorage-independent growth. CSQT-2 and MHCC97-H cells were plated in 1.0% Agar with 20% FBS complemented for 2 weeks to test the formation of colonies. (**D**) Cell migration analysis of CSQT-2 and MHCC97-H. Both cells were set for modified Boyden chamber assay to test the migratory ability.

After several passages of *in vitro* culture, we performed a series of assays to characterise the CSQT-2 cells *in vitro*. The cell growth of CSQT-2 cells was tested using the MTT assay with the MHCC97-H cells as a positive control (Figure 2A). It was found that the CSQT-2 cells had a shorter double time *in vitro* compared with MHCC97-H cells, which have been known to be fairly aggressive. The rapid cell growth in the CSQT-2 cells may be attributed to lower cell apoptosis and more rapid cell cycles. As shown in Figure 2B, about 50% of the cells were in the S-G2-M phases (Figure 2B). Moreover, the CSQT-2 cells showed stronger anchorage-independent growth abilities (Figure 2C). In the migration assay, it was shown that the migratory capacity of CSQT-2 cells surpassed that of the MHCC97-H cells (Figure 2D). Taken together, these results indicated that CSQT-2 cells could be more intensely metastatic *in vivo*.

### Characteristics of CSQT-2 cell line *in vivo*

**Figure 3:**
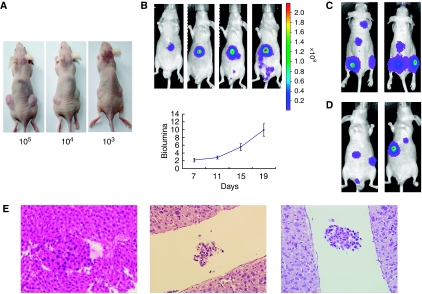
*In vivo* assay of CSQT-2 cell line. (**A**) Subcutaneous growth of CSQT-2 tumour. 100 000, 10 000 or 1000 cells of CSQT-2 were inoculated at the flanks of nude mice. Three mice were used in each group with different cell numbers. (**B**) Orthotropic growth of CSQT-2 tumour labeled with luciferase. CSQT-2 cells labeled with luciferase were first injected into the flank of nude mice. After subcutaneous tumour appeared, it was harvested and inoculated into the liver of fresh nude mice. The orthotropic tumour growth was measured by luciferase activity detection by IVIS system. (**C**) Metastatic growth of CSQT-2 tumour via cardiac injection. 500 000 cells of CSQT-2 were injected into the left ventricle of nude mice for metastasis. Shown was the metastasis 4 weeks post injection. (**D**) Metastatic growth of CSQT-2 tumour via intra-spleen injection. 500 000 cells of CSQT-2 were injected into the spleen of nude mice for metastasis. Shown was the metastasis 4 weeks post injection. (**E**) PVTT formed by CSQT-2 orthotropic tumour. The mouse livers in (**B**) were harvested for fixation and H&E staining to detect whether PVTT was formed. Three typical PVTT sections were shown.

Following the *in vitro* study, the *in vivo* behaviour of CSQT-2 cells was examined. In nude mice, CSQT-2 cells showed stronger subcutaneous tumourigenicity ability (Figure 3A). By labelling CSQT-2 cells with the Luc2 reporter gene, we successfully detected the orthotropic tumour formation in real-time. As shown in Figure 3B, CSQT-2 cells could generate HCC in immunodeficient mice. Next, to evaluate the metastatic potential of CSQT-2 cells, we performed intra-cardiac injections in nude mice, and metastatic lesions developed in the bone and lymph nodes (Figure 3C). Similarly, 3 weeks after intra-spleen injection of CSQT-2 cells, liver metastasis could be found (Figure 3D). PVTT is known to be frequently associated with the progression of HCC. In our study, orthotropic implantation of CSQT-2 tumours in the liver of nude mice generated PVTT sites in the portal vein systems of the host (Figure 3E). All of the above showed that the CSQT-2 cells recapitulated the clinical features of malignant HCC.

### Identification of the expression of tumour-initiating cell markers in CSQT-2

**Figure 4:**
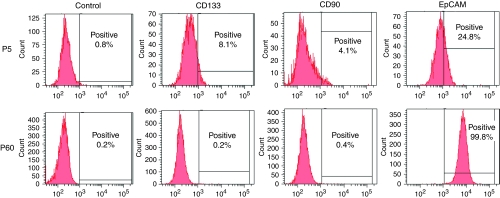
Tumour-initiating cell marker expression of CSQT-2 cell. The expression of CD133, CD90 and EpCAM were tested via FACS in both P5 and P60 generation of CSQT-2.

It has been reported that HCC probably originated from a few cancer cells that expressed certain tumour-initiating (TIC) markers such as CD133, CD90 and EpCAM. It was found that in the early passages, a small portion of the primary CSQT-2 cells expressed CD133, whereas CD133-positive cells (Figure 4A) or CD90 cells (Figure 4B) could not be detected after 30^+^ passages. In contrast to CD133 and CD90 expression, EpCAM positivity in CSQT-2 cells dramatically increased with more passages (Figure 4C).

### Identification of metastasis-related genes in CSQT-2

**Figure 5:**
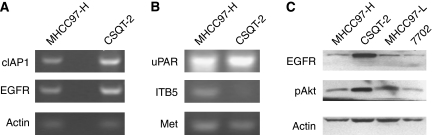
Metastasis-related gene expression of CSQT-2 cell line. (**A**) and (**B**) both MHCC97-H and CSQT-2 cells were harvested for RNA purification and then RT–PCR targeting EGFR, cIAP1, Met, uPAR, ITB5 and *β*-actin. (**C**) Cells were harvested for western blot for EGFR and phosphorylated Akt protein.

Cancer metastasis is a complicated process facilitated by the abnormal expression of metastasis-related genes, such as those involved in cell proliferation, apoptosis and migration. EGFR was dramatically overexpressed in CSQT-2 cells, which may contribute to the rapid tumour growth (Figure 5A and C). Akt, a key regulator of cell survival, was also hyperphosphorylated in CSQT-2 cells (Figure 5C), which probably led to the suppression of apoptosis. Consistent with Akt hyperactivation, cIAP1 expression was also upregulated in CSQT-2 cells. The HGF–cMet cascade was of great importance in controlling HCC cell proliferation as well as metastasis. We found that the expression of cMet was as high as that of MHCC97-H cells (Figure 5B). In addition, we tested other metastasis-related genes such as uPAR and some Integrins. Altogether, many other metastasis-related genes were found to show dysregulation in CSQT-2 cells (Figure 5).

## Karyotype of the CSQT-2 cell line

**Figure 6:**
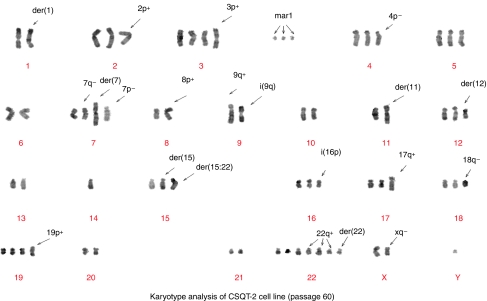
Karyotype analysis of CSQT-2 cell line (passage 60). The karyotype of the CSQT-2 cell line for abnormalities in both the number and structure in passage 60.

With the help of G-banding staining technology, we analysed the karyotype of the CSQT-2 cell line for abnormalities in both the number and structure (Figure 6). The number of chromosomes in this cell line ranged from 58 to 73 with a median of 67. At least three significant abnormalities, add(3) (qter → pter::?), der(4)t(qter → zp14::?) and add(19)(qter → pter::?) were found invariably in all cells (30/30), In addition, two other variations, add(15)(?::pter → q24::?) and add(18)(pter → qter::?) were found in most cells (29 of 30). Some other alterations, 1pter → p12::16qter → pter, add(2)(qter → pter::?) and add(7)(?::pter → qter::?) were also found frequently (Tables 1 and 2).

## Discussion

Prevention and effective control of metastasis are essential to HCC therapy, Therefore, it is urgent that we improve our understanding of the molecular mechanisms of HCC progression and metastasis (Tang *et al*, 2004; Wang *et al*, 2009). As PVTT formation is a common event in HCC metastasis, it is reasonable and worthwhile to conduct studies of PVTT-related HCC. For example, although the significant relationship between PVTT formation and HCC metastasis has been well documented by many groups, whether PVTT formation contributes to intrahepatic as well as distant metastasis of HCC is still not known (Qiu *et al*, 2008). Alternatively, it is also possible that PVTT is merely a consequence of HCC metastasis (Aldrighetti *et al*, 2009; Matsumoto *et al*, 2009). Another interesting question is how different the PVTT cells are from their primary ancestors, or how divergent they are compared with primary HCC cells (Chen and Huang, 2005). However, if a suitable cell model is not available, it is very difficult to investigate these questions.

To our knowledge, CSQT-2 is the first HCC portal vein tumour thrombus-originated cell line, which showed a relatively high metastatic potential. More importantly, this cell line was able to generate PVTT in nude mice after orthotropic inoculation. The fact that CSQT-2 is able to generate PVTT greatly facilitates our research of PVTT progression. More importantly, it has allowed us to examine whether the formation of PVTT occurs before extravascular metastasis, which could shed light on the significance of PVTT. In addition, we could investigate whether extravascular metastasis of HCC is affected by blocking PVTT formation. Moreover, as is well-known, the theory of cancer stem cells argues that a cancer mass is built up by a relatively tiny portion of the total tumour, called cancer stem cells or TIC (Guglielmi *et al*, 1998; Bansal and Banerjee, 2009; Kawasaki *et al*, 2009; Tu *et al*, 2009). The previous research on HCC TICs found that we could separate or enrich HCC TICs through specific staining for cell surface markers, such as CD133, CD90 and EpCAM (Yang *et al*, 2008a, 2008b; Yamashita *et al*, 2009; Yeh *et al*, 2009; Yoshikawa *et al*, 2009; Zhu *et al*, 2009). It is intriguing that the CSQT-2 cell line showed no CD133 or CD90 positivity, even though the corresponding primary cells contained apparent CD133- and CD90-positive subpopulations, this suggested that these two markers might not be critical in our cell line. Surprisingly, the whole population of CSQT-2 cells in the cell line was EpCAM positive. This might explain the strong xenografting ability of CSQT-2 in nude mice (Yamashita *et al*, 2008). Furthermore, such observation also raised the possibility that the TIC marker expression might be different between primary and portal vein invasive HCC cells. If confirmed, this would undoubtedly alter our interpretation of the cancer stem cell theory, and we can establish the HCC cell line from the primary HCC and the paired PVTT cell line. Once we succeed, the difference as well as the similarities between these two types of cell lines could be compared, this will not only broaden our knowledge of the progression of HCC and PVTT, but it will also allow the study of the mechanism through which HCC can progress to PVTT.

## Figures and Tables

**Table 1:** Appearing frequency of abnormal chromosomes in 30 karyotype analysed (passage 60)

	**Extra chromosomes**							**Abnormal chromosomes (markers)**						
Chromosome	−Y	+2	+3	+3 × 2	4	5	6	M3	M4	M5	M6	M7	M8	M9
Frequency	9	16	16	13	23	16	22	18	27	8	28	30	30	28
Chromosome	7	+7 × 2	−8	−9	10	+10 × 2	+10 × 3	M10	M11	M13	M14	M15	M16	+Mar16 × 2
Frequency	15	9	10	9	6	16	6	24	28	16	25	21	10	8
Chromosome	−11	12	−13	−13 × 2	−14	15	+15 × 2	M18	M19	M20	M21	M22	M23	M24
Frequency	11	18	19	7	16	21	9	12	6	24	9	29	10	18
Chromosome	16	+16 × 2	+16 × 4	17	+17 × 2	18	+18 × 2	M25	M26	M27	M28	M29	M30	+Mar31 × 2
Frequency	4	20	6	15	14	9	9	26	10	29	15	30	19	20
Chromosome	+18 × 3	19	+19 × 2	20	+20 × 2	21	+22 × 2	+Mar31 × 3	+Mar1 × 2	+Mar2	M12	M17		
Frequency	12	18	6	16	8	10	18	10	25	7	3	2		
Chromosome	+22 × 3	+Mar1 × 2	+Mar2	+Mar16 × 2	+Mar31 × 2	+Mar31 × 3								
Frequency	10	25	7	10	20	10								

Appearing frequency of abnormal chromosomes exceeded 50% of karyotype analysed.

**Table 2:** Abnormal chromosomes for CSQT-2 cell lines

**Chromosomes**	**Markers**
M1	dmin
M3(xq−)	del(x)(pter → q26:)
M4(der(1))	1pter → p12::16qter → pter
M5(1q−)	del(1)(pter → q23:)
M6(2p+)	add(2)(qter → pter::?)
M7(3p+)	add(3) (qter → pter::?)
M8(4p−)	der(4)t(qter → p14::?)
M9(6q+)	add(6) (pter → qter::?)
M10(7q−)	del(7)(pter → q33:)
M11(der(7))	add(7)(?::pter → qter::?)
M12(7p−)	del(7)(qter → p13:)
M13(8p+)	add(8)(qter → pter::?)
M14(9q+)	add(9)(pter → qter::q11 → qter)
M15(i(9q))	i(9)(q10)
M16(der(10))	add(10)(?::pter → qter::?)
M17(10p−)	del(10)(qter → p12:)
M18(i(10q))	i(10)(q10)
M19(der(11))	add(11)(pter → qter::?)
M20(der(12))	der(12)t(qter → p12::?)
M21(i(14q))	i(14)(q10)
M22(der(15))	add(15)(?::pter → q24::?)
M23(der(15;22))	der(15)t(15qter → pter::22q11 → qter)
M24(i(16p))	i(16)(p10)
M25(der(16))	add(16)(pter → qter::?)
M26(17q+)	add(17)(pter → qter::?)
M27(18q+)	add(18)(pter → qter::?)
M28(18q−)	del(18)(pter → q21:)
M29(19p+)	add(19)(qter → pter::?)
M30(der(22))	der(22)(?::pter → q12:)
M31(22q+)	add(22)(pter → q11::?)
